# Optimizing Management of Patients with Adult T Cell Leukemia-Lymphoma

**DOI:** 10.3390/cancers7040893

**Published:** 2015-11-25

**Authors:** Jean A. Yared, Amy S. Kimball

**Affiliations:** Marlene and Stewart Greenebaum Cancer Center, University of Maryland School of Medicine, 22 S. Greene Street, Baltimore, MD 21204, USA

**Keywords:** adult T cell leukemia-lymphoma, ATL, ATLL, allogeneic, HTLV-1, transplantation, chemotherapy, antiviral treatment

## Abstract

Adult T cell leukemia-lymphoma is a rare disease with a high mortality rate, and is challenging for the clinician. Early allogeneic stem cell transplant can confer durable remission. As novel therapeutic agents become available to treat T cell malignancies, it is increasingly important that medical oncologists, hematologists, and hematopathologists recognize and accurately diagnose adult T cell leukemia-lymphoma. There is no uniform standard of treatment of adult T cell leukemia-lymphoma, and clinical trials remain critical to improving outcomes. Here we present one management approach based on the recent advances in treatment for adult T cell leukemia-lymphoma patients.

## 1. Introduction

Adult T cell leukemia-lymphoma (ATL) is an aggressive lymphoid neoplasm that occurs only in patients with human T-lymphotropic virus, type I (HTLV-1) viral infection [1]. Two HTLV-1 genes play key roles in ATL. The viral Tax transactivator protein acts early, after infection, causing overexpression of cellular and viral genes, and leading to proliferation of HTLV-1-infected T-cell clones. Tax is likely essential to the establishment of ATL. Tax expression is not, however, detected in all cases of circulating ATL cells, suggesting that Tax does not exclusively drive proliferation in the disease state. HTLV-1 basic Zip factor (HBZ) gene expression is uniformly detected in patient ATL cells and, thus, appears critical; inhibition of HBZ function is of interest as a treatment strategy. The biology of ATL is the subject of fascinating ongoing research and is beyond the scope of this clinically-oriented review.

ATL develops in a small fraction (4% to 5%) of HTLV-1 infected patients several decades after primary infection [2,3]. During the long latency period the HTLV-1 virus undergoes genetic changes, with paired blood and nodal samples from ATL patients often showing different genome profiles [4]. The incidence of ATL is strongly correlated to the seropositivity of HTLV-1 of the birth place, and common in people originating from Japan, the Caribbean basin, Central and South America, Western Africa, Iran and Southeast USA [5,6]. Only 140 US cases have been documented in the SEER database between 1993 and 2008. As it is rare and has a variable clinical presentation, ATL is likely under-diagnosed in the USA. HTLV-I infects an estimated 10 to 20 million people worldwide and is primarily transmitted by breast feeding, although spread via blood transfusion—although very rare nowadays, sharing of needles, and sexual intercourse also occurs [7]. Much of the clinical data has been obtained in Japan or the Middle East, where the viral subtype compositions differ from the Caribbean. Outcomes may vary based on region of origin, with Caribbean ATL reported to have lower survival among patients diagnosed in the USA [8] and socio-economic factors. Patients with ATL present with chronic or acute disease, with a remarkable array of organ involvement seen. This heterogeneous presentation of ATL contributes to the clinical challenges of diagnosing and caring for ATL patients. The most common presentations of ATL are the acute and lymphoma type [9] which are highly aggressive and have a poor prognosis with survival measured in 6–10 months even with aggressive chemotherapy [10]. Recent work suggests that a subset of ATL patients may benefit from antiviral therapy alone without chemotherapy [11]. Allogeneic stem cell transplant can significantly prolong survival. There is no standard treatment for ATL; guidelines recommend that patients be enrolled in clinical trials whenever possible.

## 2. Recognizing ATL

ATL is a malignancy of mature T-cells, and is notable for the lack of a uniform clinical presentation. ATL can present as ATL lymphoma, with enlargement of lymph nodes, can involve only extranodal sites, or can present as leukemia. Patients often have marked hypercalcemia as the presenting sign.

The first challenge to the clinician is to consider the possibility of ATL in new cases of mature T cell malignancy. The diagnosis is easily established in leukemic ATL, where patients have circulating clonal T-cells. These ATL cells often have characteristic multilobed nuclei, after which they are called “flower cells”. Alternately, the cells may have cerebreform nuclei reminiscent of Sezary cells.

A biopsy of lymph nodes involved with ATL lymphoma can resemble peripheral T-cell lymphoma not otherwise specified (PTCL-NOS), anaplastic large cell lymphoma (ALCL), angioimmunoblastic T-cell lymphoma (AITL), or mycosis fungoides/Sezary syndrome (MF). It is characterized by medium sized lymphocytes with condensed chromatin and irregular hyperlobated nuclei (“clover leaf” or “flower cells”). As in other T-cell malignancies, the immunohistochemistry of ATL is somewhat variable, and clinical context remains an element contributing to accurate diagnosis. The clinician should work collaboratively with the hematopathologist to review findings and consider the diagnosis. Evaluation of HTLV-1 serology is an appropriate first step in evaluating the diagnosis, and we test every new PTCL patient for HTLV-1.

ATL expresses mature T cell markers; the cells are generally CD2, CD5, HLA-DR, and TCRαβ positive, with aberrant loss of CD7, and often with low CD3. ATL can often be distinguished from other T-cell lymphomas by its strong uniform expression of CD25/C-C chemokine receptor type 4 (CCR4). ATL cells are a malignant counterpart of regulatory T cells, and are usually CD4 positive and CD8 negative, though this is not always true. While CD25 expression helps secure an ATL diagnosis, CD25 is also expressed in Sezary syndrome and T cell prolymphocytic leukemia. ATL cells can express CD30, and can express CD15. Given the immunohistochemical variability of the transformed T-cells, detection of HTLV-1 positivity is essential and allows the diagnosis of ATL [12].

ATL cells have varied and complex genomic changes, arising during the long latency before development of malignancy. There are no characteristic translocations that contribute to the diagnosis. Nucleic acid hybridization or Southern blotting detects HTLV-1 proviral sequence in the malignant cell, though in most cases HTLV-1 Ab is detected and is sufficient to support a diagnosis. Detection of intracellular viral DNA may be useful in equivocal cases.

## 3. Shimoyama Classification

Once a pathologic diagnosis of ATL is made, patients should be classified into acute, lymphoma, chronic unfavorable, chronic favorable, or smoldering subtypes [13,14]. (Table 1) Classification directs management. Circulating cell counts, LDH, calcium level, and sites of involvement are the first factors used to make the classification. In cases with intermediate cell count levels classification can become complex, and depends upon sites of ATL lesions [15] (Table 1).

**Table 1:** The Shimoyama Classification.

	Acute ^ (Leukemic)	ATL Lymphoma	Chronic Unfav *	Chronic Fav	Smoldering	Pre-ATL
Anti-HTLV-1 Ab	+	+	+	+	+	+
Circulating ATL cells	+	-	+	+	+	+
Lymphocytosis (ALC > 4000)	Variable	No	Yes	Yes	No	No
Circulating abnormal lymphocytes	Variable	≤1%	Variable	Variable	≥5% or <5% if ATL lesion(s) in the skin and/or lung	
LDH	Variable	Variable	<2× ULN, or *	normal	≤1.5 ULN	normal
Calcium level	Variable	Variable	<11.0	normal	normal	normal
Rash	Variable	Variable	Variable	Variable	Variable	No
Lymphadenopathy	Variable	>1.5 cm	Variable	Variable	No	No
Organomegaly	Variable	Variable	Mild	Mild	No	No
BUN			>ULN	NL		
Albumin			<LLN	NL		
CNS involvement	+/−	+/−	No	No	No	No
Bone lesions	+/−	+/−	No	No	No	No
Ascites	+/−	+/−	No	No	No	No
Pleural effusion	+/−	+/−	No	No	No	No
GI tract	+/−	+/−	No	No	No	No

^ Acute type ATL is an exclusion diagnosis after ruling out other types of ATL; * Unfavorable Chronic subtype is distinguished from favorable by at least one of: high LDH, high BUN or low albumin.

ATL most commonly presents as a leukemia (termed acute ATL) or a lymphoma; these are collectively referred to as aggressive ATL. Acute ATL is diagnosed when HTLV-1-positive patients have >4000 circulating lymphocytes, of which >/= 5% are clonal T-cells, and either LDH is more than two times the upper limit of normal or Ca is >/=11.0 mg/dL.

ATL lymphoma patients have enlarged lymph nodes with demonstrated ATL involvement, and low or undetectable circulating ATL cells. To fulfill criteria for ATL lymphoma there must be less than 4000 clonal T cells per microliter, and less than 1% of the circulating lymphocytes may be ATL cells. The inclusion of a low circulating cell count to define ATL lymphoma type is questioned by some, who suggest that any patient with a nodal mass requires systemic cytotoxic chemotherapy [11].

In both of the aggressive forms, calcium levels are often high, there can be visceral involvement, and there may be B symptoms. Patients with aggressive ATL require treatment upon diagnosis.

The so-called indolent ATL subtypes were historically not treated on diagnosis, but this is changing. It is, thus, increasingly important to accurately classify these cases.

Several clinical situations are defined as chronic ATL. When there is a lymphocytosis arising from circulating ATL cells and LDH and calcium levels are below those fulfilling criteria for leukemic acute ATL, a case is classified as chronic unfavorable. In smoldering ATL circulating ATL cells are detected at less than the threshold for leukemia or chronic ATL, and laboratory values are within normal limits, except for LDH, which can be up to 1.5× ULN.

## 4. Baseline Laboratory Studies and Staging of ATL

The variety and heterogeneity of organ involvement in ATL is such that standard lymphoma staging studies, and additional ATL-specific staging studies should be done on diagnosis (Table 2). The disease has a predilection for bone and skin; therefore, a full skin exam is performed on all patients and a skeletal survey is ordered on select patients with bone symptoms, fractures or unexplained elevated alkaline phosphatase. A bone marrow aspirate, biopsy, and cytogenetics are performed at diagnosis. ATL can involve cardiac tissue; therefore all patients have a baseline ECHO or MUGA study done, and a baseline troponin level is drawn. Additional baseline laboratory testing includes a complete metabolic panel, calcium, LDH, uric acid, CBC with differential, peripheral blood flow cytometry, and HTLV-1 quantitative DNA. Soluble IL2 receptor (sIL2R) is a marker of disease, and is evaluated at baseline. Baseline blood CMV serology is checked, to allow monitoring for CMV reactivation should the patient undergo allogeneic stem cell transplantation (allo-HCT). The blood G6PD level is checked at baseline as a low G6PD level may influence tumor lysis syndrome prophylaxis and treatment choices.

**Table 2:** Baseline evaluation of newly diagnosed ATL patient.

**Exam:**
Physical Examination including full skin exam and assessment of adenopathy and hepatosplenomegaly
**Imaging:**
PET/CT
Skeletal survey (for assessment of bone lytic lesions)
ECHO or MUGA (to rule-out cardiac involvement and before initiating anthracycline-based chemotherapy)
**Laboratory:**
CBC with differential
Chemistry panel including Calcium (assessment of hypercalcemia)
Peripheral blood smear (for assessment of circulating lymphocytes with “flower-like” nuclei)
LDH, uric acid
HTLV-1 quantitative DNA PCR
Troponin
HLA typing
CMV serology
G6PD
**Other:**
Bone marrow aspirate, biopsy, and cytogenetics
Transplantation evaluation

All newly diagnosed ATL patients have a PET/CT done to identify baseline sites of disease [16]. When a patient has ATL lymphoma, the staging follows standard lymphoma staging practice.

Allogeneic stem cell transplant can confer long term remissions; therefore, an HLA typing on every new, fit, acute ATL patient is suggested to initiate a transplant evaluation.

ATL management is directed by ATL subtype. New ATL cases must be classified as acute, lymphoma-type, chronic unfavorable, chronic favorable, or smoldering as above.

## 5. Treatment

**Figure 1:**
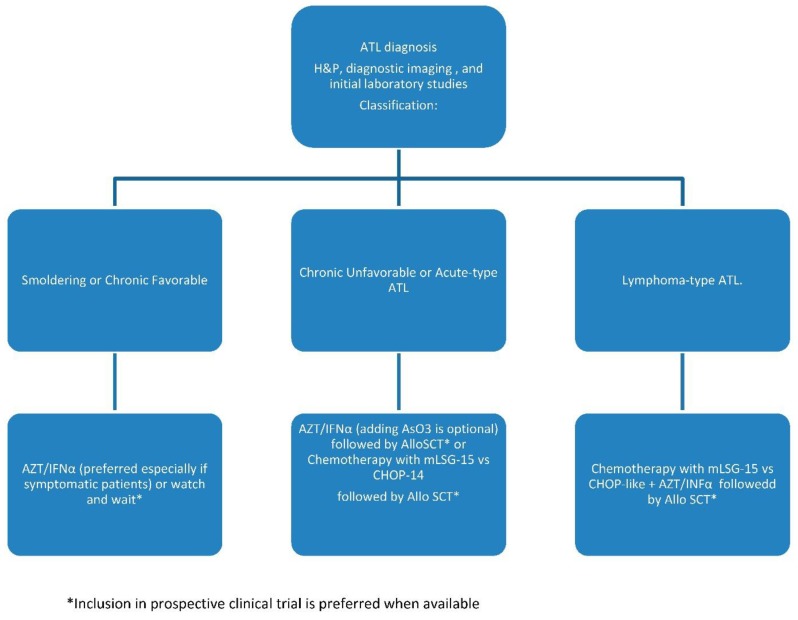
ATL treatment algorithm.

There is no uniform standard of care for ATL. Before the revised January 2015 guidelines, the NCCN recommended only CHOP or CHOP-like therapy for aggressive ATL. We have adopted a treatment approach that is described below (Figure 1).

All ATL patients are referred for clinical trial therapy when such therapy is available. When there is not a clinical trial option, a reasonable approach is described below. Treatment options have sometimes been influenced by insurance authorization, which varies between insurers, due to the paucity of information to guide treatment.

### 5.1. Smoldering and Chronic Favorable

Patients with smoldering or chronic favorable ATL survive a median of approximately four years when untreated, as was the recent standard of care [17]. *In vitro* studies suggest that zidovudine (AZT) therapy given together with interferon-alpha (IFNα) leads to apoptosis of HTLV-1-infected cells [18]. Though the mechanism, of AZT and IFNα is debated, the combination usually has clinical activity in treating ATL. We refer to AZT and IFNα therapy as “antiviral therapy” in this text.

When antiviral therapy was given on diagnosis, 17 of 29 treated patients with chronic or smoldering ATL were alive at five years [11]; therefore, treatment of smoldering and chronic favorable patients upon diagnosis is suggested. The dosing of interferon and choice of and dosing of antiviral have not been standardized. One approach is to initiate INFα 2b (IntronA), beginning at 5 MU subcutaneously daily, and Zidovudine (AZT) at 300 mg tid. CBC, LFTs and Cr are monitored at least weekly. If the platelet count falls below 50,000, if ANC falls below 500 or if LFTs rise to greater than 2.5× ULN interferon is reduced to 3 MU daily and AZT to 300 mg bid. IFNα therapy is given for 8 to 12 weeks when patients are responding.

On completion of IFNα therapy, AZT at 300 mg three times daily is continued indefinitely. Patients are followed clinically, with flow cytometry and HTLV-1 viral load monitored every three months.

### 5.2. Chronic Unfavorable and Acute ATL

Patients with acute or with chronic unfavorable require systemic therapy. Historically these patients have done poorly and there has not been a clearly preferred treatment approach. This, however, is changing.

ATL cells express P-glycoprotein and other multidrug resistance proteins (MRP and LRP) [10]. Conventional chemotherapy therefore is not always effective in ATL. The most effective cytotoxic chemotherapy regimen for acute ATL is modified LSG15 (mLSG15). This gives a 40% complete response rate with a median survival time of only approximately one year in acute ATL [19]. The therapy is arduous, requires growth factor support, and confers a 98% risk of grade three or four neutropenia, 74% grade three or four thrombocytopenia and 32% risk of serious infection [19].

While retrospective, an analysis of acute ATL patients treated with frontline IFNα and AZT found a survival advantage as compared to those treated with frontline chemotherapy [11]. When initial treatment with IFNα and AZT were given, 28% of acute ATL patients were alive at five years, compared to 12% of acute ATL patients alive at five years when chemotherapy was given before antiviral therapy [11]. Thus, starting treatment with an antiviral regimen for acute leukemic ATL patients is a reasonable approach.

Responses to IFNα and AZT in acute ATL patients are sustained for two months up to nearly three years [20]. The addition of arsenic trioxide (AsO3) to INFα and AZT (AIZ) has been tested as frontline therapy for patients with chronic ATL. For chronic ATL, AIZ gave a 70% complete response rate and 100% overall response rate [21]. Responses to AIZ occurred after 2–4 weeks on therapy; the duration of response in chronic ATL is not yet reported.

As AIZ is comparatively nontoxic and active therapy, in relation to mLSG15, AIZ combination as a first-line treatment for newly diagnosed acute leukemic ATL patients is an acceptable approach. As such, a baseline EKG is done, and the QTc interval is required to be <460 ms for treatment. Potassium and magnesium levels are monitored at least twice weekly and repleted as needed. AsO3 therapy is given at 10 mg/day as an outpatient, Mon-Fri, for up to six weeks. IntronA at 5 MU subcutaneously daily and AZT at 300 mg two times daily are given concomitantly through AsO3 therapy. CBC, LFT’s and Cr are monitored at least weekly. If the platelet count falls below 50,000, if ANC falls below 500 or if LFT’s rise to greater than 2.5× ULN, Interferon is reduced to 3 MU daily, then 3 MU TIW if needed. Upon completion of AsO3, patients are maintained on IFN to complete eight or 12 weeks and AZT indefinitely.

If patient factors do not allow administration of frontline AsO3 therapy to acute ATL patients, antiviral therapy, without AsO3, is pursued. INFα and AZT are most effective when offered as an initial treatment, and at active doses, rather than on relapse after chemotherapy. Frontline IFNα and AZT have a 57%–85% response rate in acute ATL, with short responses [22,23]. Therefore, initiating antiviral therapy before conventional chemotherapy with close monitoring in acute ATL patients is rational. If conventional chemotherapy is initiated, we attempt to continue IFNα and AZT during chemotherapy, as tolerated by blood counts and liver function.

Outcomes for acute ATL patients treated with frontline AIZ are not yet reported and duration of response is not known. When a stem cell donor is available we, thus, proceed to transplant.

When antiviral therapy for acute ATL is not effective, conventional chemotherapy may be pursued as discussed below.

### 5.3. ATL Lymphoma

As for acute leukemic ATL, our first consideration in treating ATL lymphoma patients is evaluation of clinical trial opportunities.

In contrast to acute ATL, however, conventional cytotoxic chemotherapy appears to have a critical role in prolonging survival of ATL lymphoma patients [11]. Inclusion of IFNα and AZT with initial chemotherapy treatment improves outcomes, conferring a longer survival than does chemotherapy alone [11]. Therefore, a combined approach is promising.

Conventional Chemotherapy: The most active ATL regimen is LSG-15 [19]. In a randomized phase three trial modified LSG-15 (mLSG-15) therapy gave a 40% compete response rate, with 28% PFS at one year. This compared favorably to CHOP-14 which gave a 25% RR and 16% one year PFS [19]. Fit acute ATL patients can be offered a variant of LSG-15. LSG-15 variant is composed of agents available in the US: carmustine is substituted for ranimustine [24] and vinorelbine is substituted for vindesine, though vincristine substitution has also been recommended [24]. LSG-15-like chemotherapy can be given outpatient and consists of VCAP on day one (vincristine 1 mg/m^2^, cyclophosphamide 350 mg/m^2^, doxorubicin 40 mg/m^2^ and prednisone 40 mg/m^2^). On day eight ACP (doxorubicin 30 mg/m^2^, carmustine 60 mg/m^2^ and prednisone 40 mg/m^2^) is given. On day 15 VECP (vinorelbine 25 mg/m^2^, etoposide 100 mg/m^2^, carboplatin 250 mg/m^2^ and prednisone 40 mg/m^2^) is started. Etoposide and prednisone are given, at the day 15 doses, on days 16 and 17 as well.

This mLSG-15-like therapy repeats every four weeks or upon recovery of neutrophil counts. Up to six cycles of LSG-15-like therapy are given. Intrathecal prophylaxis with 15 mg methotrexate and 40 mg of cytarabine is given three times during therapy, before cycles two, four, and six. Blood counts are checked twice weekly and GCF support is given between treatments when ANC is <1000.

The mLSG-15 regimen is quite myelosuppressive, especially that carmustine is substituted for ranimustine. For day one therapy ANC is required to be above 1200, for day eight, above 1000, and for day 15, above 500. Dose delays due to neutropenia are frequent even with growth factor support. Tsukasaki *et al* gave cycles five and six on a mean of a 42 days cycle rather than the planned 28 day cycle. If a patient experienced a serious infection, doses were reduced to 75%, after a second serious infection patients were taken off chemotherapy. Tsukasaki and his colleagues treated 57 patients and completed planned treatment in only 32% [19].

Progressive disease in 40% of patients was the primary reason for discontinuing therapy in the Japanese trial. We thus take patients to transplant as soon as a transplant plan is in place after initiating chemotherapy.

INFα and AZT can be initiated with chemotherapy and continued as tolerated by neutrophil and platelet counts. When given concurrently, we give IFNα 3 million units daily and AZT 300 mg bid [25].

When mLSG-15-like therapy is not feasible, dose-adjusted EPOCH or CHOP combination chemotherapy are considered as these are well described in ATL [19,26]. Dose-adjusted EPOCH is associated with a 15%–35% complete response rate in ATL. CHOP has a 25%–55% response rate in lymphomatous ATL cases. The inclusion of antiviral therapy prolongs survival in lymphoma-type, though overall survival with the combined therapy remains short at 20% 3 year survival [25]. At least three cycles of intrathecal chemotherapy prophylaxis is considered, after LSG-15.

In ATL lymphoma, response is assessed with clinical exam, and interim CT scans after cycle two and after cycle four are suggested if there is suspicion of refractory or progressive disease.

Our ATL lymphoma patients proceed to allogeneic stem cell transplant in first remission. If a transplant plan is not in place we continue IFNα and AZT and close monitoring.

Other potentially active chemotherapy regimens that we consider include Gemcitabine Oxaliplatin, DHAP, or pralatrexate. These three regimens are offered on the control arm of a current NIH trial evaluating the experimental agent anti-CCR4 (KW-0761). Consideration for alemtuzumab therapy can be given, although it was withdrawn from the market in the US in 2012 and, therefore, needs to be obtained through the company for compassionate use.

All patients are evaluated for allogeneic stem cell transplant in first remission. We proceed to transplant as soon as a donor is available provided the disease remains responsive to treatment.

### 5.4. Single Site of Lymphomatous, or Bone, Disease

ATL can rarely present with a single bone or skin lesion as the only detectable disease. Radiation can control ATL [27]. In these cases, external beam photon or electron therapy, up to 60 Gy, is considered. Following radiation treatment, INFα and AZT is suggested, because the majority of patients suffer local or distant relapses [27].

### 5.5. Pre-ATL

Pre-ATL has recently been described, but is not a part of the Shimoyama classification. When pre-ATL cases are identified, we monitor closely without treatment.

## 6. Response Assessment

Patients with ATL lymphoma are assessed according to standard lymphoma response criteria [16]. CT scans after cycle two and four of chemotherapy are suggested if there is suspicion of refractory or progressive disease, and an end of treatment PET-CT is performed. ATL response assessment requires a high index of suspicion and ongoing clinical monitoring for the development of new skin or bone lesions throughout therapy.

Though not standardized, in cases where circulating disease is detectable at baseline, peripheral blood flow cytometry can be performed after 15 days of AIZ therapy, or on count recovery prior to cycles three and five of traditional chemotherapy. For research purposes, we also monitor HTLV-1 quantitative PCR before cycles three and five, when detectable at baseline.

## 7. Prophylaxis against Opportunistic Infection

ATL is immunosuppressive; infection was the presenting sign of ATL in a third of Japanese patients with chronic or smoldering ATL [13]. ATL patients are treated with prophylactic sulfamethoxazole/trimethoprim and acyclovir.

Antifungal prophylaxis is suggested for patients undergoing preparation for allogneic stem cell transplant. CMV status is assessed at baseline and CMV DNA levels are monitored through chemotherapy.

Systemic infection by strongyloides can occur in Carribean ATL patients who may benefit from prophylactic ivermectin or albendazole [28].

## 8. Hematopoietic Cell Transplantation

Aggressive ATL mortality is now significantly decreasing and this trend might be associated with the widespread of allo-HCT [29], but not autologous hematopoietic cell transplantation [30]. Allo-HCT is associated with significant treatment-related mortality but offers a potential graft-*versus*-leukemia effect [31,32,33,34], and grade I-II acute graft-*versus*-host disease (GVHD) and limited or extensive chronic GVHD were associated with improved overall survival (OS) demonstrating the actual existence of graft-*versus*-ATL (GVL) effects [35]. After allo-HCT, virus load significantly decreased in some patients [32,36], suggesting that anti-HTLV-1 immune response is enhanced in these patients. In fact, HTLV-1 Tax-specific cytotoxic T cell clones were detected in both peripheral blood and bone marrows over three years in patients after Allo-SCT [37]. The largest retrospective data is available from Japan describing 386 patients with ATL who underwent allogeneic HCT with a three-year survival up 45% [38] that is better than the predicted 25% for patients treated with chemotherapy only [39]. Patients who developed mild or moderate (grade1/2) GVHD had superior overall survival than those without grade 3/4 GVHD [40]. Male sex, high level of serum soluble interleukine-2 receptor at HCT, and non-complete remission at HCT predict poor outcome after HCT [41]. No significant difference in OS between a myeloablative conditioning regimen (MAC) and a reduced intensity conditioning regimen (RIC) was observed, with a trend of better overall survival (OS) in older adults treated with RIC [42]. The preferred donor is a HLA-matched related or unrelated donor who is HTLV-1 negative proved by the absence of HTLV-1 antibodies or antigens in blood because of the risk of donor-derived ATL [43,44]. Bone marrow or peripheral blood can be used as source for hematopoietic stem cells; cord blood transplantation in ATL is still considered experimental and carries high non-relapse mortality (46%) with a relative low two-year OS rate (20%) [45]. GVHD prophylaxis classically includes intermittent methotrexate in association with a calcineurin inhibitor such as cyclosporine or tacrolimus or using the Dana-Farber combination of tacrolimus and sirolimus [46] knowing that the latest agent has mTOR inhibition properties that induces senescence in adult T-cell leukemia/lymphoma and apoptosis in peripheral T-cell lymphomas [47]. Relapsed ATL after allo-SCT can be successfully treated with donor lymphocyte infusion with resulted durable remission suggesting that induction of GVL effect is real and crucial [48,49].

At our institution, high-risk ATL (chronic unfavorable-, acute-, and lymphoma-type) patients who had a favorable response to induction treatment and to whom a donor (matched related or unrelated, or partially, (7/8) matched related or unrelated, or haploidentical) is available are offered allogeneic SCT. We favor an HTLV-1 negative donor proved by the absence of HTLV-1 antibodies or antigens in blood. Donor selection and evaluation will be in accordance with our institution Blood and Marrow Transplant Program policy. The choice of a myeloablative *vs.* a non-myeloablative conditioning regimen is according to the treating physician. The MAC regimen is standard TBI-Cytoxan or high Busulfan-Fludarabine [38]. The RIC regimen is Busulfan-Fludarabine +/− TBI (2 to 4 Gy). GVHD prophylaxis will be generally with Tacrolimus + Sirolimus as detailed above.

## 9. Future Directions

A majority of cases of ATL are positive for CCR4. An anti-CCR4 monoclonal antibody, mogamulizumab (Poteligeo®) is commercially available in Japan [50] and has a 50% response rate as a single agent in phase two studies with manageable toxicities, including skin reactions [51]. This agent is currently under investigation on ongoing randomized trials in the U.S.

Several promising new agents for ATL are currently under investigation and some are now in clinical trials. Among them are brentuximab vedotin [52], proteasome inhibitors, such as bortezomib, which inhibits ATL cells growth *in vivo* and *in vitro* [53], lenalidomide [54], and IL2 fused with the diphtheria toxin targeting CD25, praletrexate, and others, which are primarily being tested in Japan.
